# The Story of the Insane from Year to Year

**Published:** 1894-01-27

**Authors:** 


### THE STORY OF THE INSANE FROM YEAR TO YEAR.

(Sixth Series.)

### WARWICK COUNTY ASYLUM.

During last year the numbers increased from 698 to 739; 275 patients were admitted, 86 were discharged recovered, 13 were discharged relieved, and 79 died. The percentage of recoveries is given in Table III. as 32·8 for the men and 63·7 for the women; both sexes, 48·8. We do not see how these percentages have been arrived at, as, taking the figures given in Table I., the recoveries stand at—males, 30·7; females, 43·7; both sexes, 38·2. Of the admissions, 41 were driven mad by hereditary influence, and 14 by drink. Cerebral diseases account for 28 of the deaths, and consumption for 11. Five of the deaths were owing to diarrhœa, dysentery, and enteritis. Dr. Miller does not attribute these to defective sanitation, and truly says that "diseases of the intestinal tract are, and always will be, prevalent among the very demented, whose depraved habits tend to set up an unhealthy condition." Dr. Miller had in the year 121 incurables, composed of congenital idiots, epileptics, paralytics, and chronic cases—certainly not a very favourable lot to work on. The weekly rate of maintenance is 8s. 5½d.

### BELFAST DISTRICT ASYLUM.

This asylum contained 691 patients on December 31st, 1892, showing an increase of twenty patients during the year. There were 216 admissions, 65 recoveries, and 47 deaths. The recoveries work out at 30·1 per cent. of the admissions, and the deaths at 7 per cent. on the average number resident, while their averages for twelve years were respectively 43·2 and 5·6. From table 17 we learn that drink caused 10 of the admissions, and hereditary influence and previous attacks account for 45. Of the deaths, 38 were owing to cerebral and spinal diseases, and only 2 to consumption, although the asylum was extremely overcrowded. This crowding will be removed when the new asylum for the county of Antrim is built, and the present asylum will then be reserved for the city of Belfast, or, as has been advised by the Irish Commissioners, the old asylum will be sold and a new one built for the city. That this would be the proper course there cannot be a doubt, and it is probable it would be in the end the most economical for the ratepayers. The Government Inspector speaks out very plainly on the evils of the present system, and when describing one of the female wards that "such a scene could not now be found in any asylum in England or Scotland," and he adds that the medical officers are in no way to blame for it. As is customary in Irish asylums the Committee of Visitors do not publish their report, but there is one from the auditor. The annual cost per head is a little over £22 10s.

### SOMERSET COUNTY ASYLUM.

The forty-fifth annual report tells us that the numbers rose from 818 to 867. The admissions reached 251, the recoveries 108, and the deaths 57. We learn from Table X that drink caused the insanity in 17 of the admissions, religion in 3, and hereditary influence in 20 only; but the cause is stated as "unknown" in 72 and "previous attacks" in 46. Of the deaths, 24 were from brain diseases, 8 from consumption, and 1 from dysenteric diarrhœa. The recoveries stand at 44·9 per cent. on the admissions, and the deaths at 6·7 on the average number resident. The asylum is insufficient for the population, and 116 are at present boarded out in other asylums; and even with this expedient Dr. Wade looks upon the position of affairs as serious, as it will be three years before the new asylum can be opened. A note to the visitors' report informs us that a tender was accepted on October 18th, 1892, for the erection of the buildings, and that the sum was £113,300. We believe the asylum is planned for about 600 patients, so that it will cost the enormous sum of £190 a bed; and this sum, large as it is, does not include roads, fences, electric light, reservoir, or water pipes. The visitors say that "Dr. Wade's endeavours to promote the efficiency of the asylum have been unremitting." The weekly cost per head is a trifle over 9s. 3d.

### VALKENBERG ASYLUM, CAPE OF GOOD HOPE.

This is a small asylum containing on January 1st, 1893, only 69 patients, and this number shows an increase of 11 during the year 1892. There were 27 admissions, six recoveries, and two deaths. The recoveries work out at 23 per cent. of the admissions, and the deaths at 3·2 on the average number resident. The effect of hereditary influence in the production of insanity is more marked even than in our asylums, for not fewer than 13 of the 27 admissions were owing to this cause. Dr. Dodds says that "public opinion, and still more, individual practice, keeps a long distance behind ascertained fact in this matter of heredity," and so it does, but we have some hope that in time the "sermons of science" will be listened to and acted on. At all events, we shall continue to point out, as we have done for the last six years in The Hospital, the terrible danger to the offspring of marriages in which either parent has a tendency to insanity. The building used at Valkenberg as an asylum is apparently quite unsuitable for its purpose, and plans for an entirely new one have been prepared. The weekly cost of maintenance is 22s. 9¾d. per head.

